# Depression, anxiety, stress, and dysmenorrhea: a protocol for a systematic review

**DOI:** 10.1186/s13643-020-01319-4

**Published:** 2020-03-26

**Authors:** Amir H. Pakpour, Farideh Kazemi, Zainab Alimoradi, Mark D. Griffiths

**Affiliations:** 1 Social Determinants of Health Research Center, Research Institute for Prevention of Non-Communicable Diseases, Qazvin University of Medical Sciences, Bahonar blv., Qazvin, 34197-59811 Iran; 2 Department of Nursing, School of Health and Welfare, Jönköping University, Jönköping, Sweden; 3 Mother & Child Care Research Center, Department of Midwifery, School of Nursing and Midwifery, Hamadan University of Medical Sciences, Hamadan, Iran; 4 International Gaming Research Unit, Psychology Department, Nottingham Trent University, Nottingham, UK

**Keywords:** Depression, Anxiety, Stress, Dysmenorrhea, Systematic review

## Abstract

**Background:**

Dysmenorrhea is one of the most common menstrual disorders and is influenced by various factors. Psychological disorders including anxiety, depression, and stress have been suggested as influencing dysmenorrhea, but previous findings are inconsistent. This study will investigate the relationship between depression/anxiety/stress and dysmenorrhea using a systematic review and meta-analysis.

**Methods:**

Online databases including *PsycINFO*, *Scopus*, *PubMed*, *Science Direct*, *ProQuest*, *ISI Web of Knowledge*, and *Embase* will be searched. Appropriate keywords and MeSH terms will be used to retrieve the journal papers published from 1990 until the end of December 2019. To improve search coverage, the reference lists of all included studies will be reviewed to find eligible papers. Inclusion criteria include the following: descriptive, cohort, case-control, and cross-sectional studies; the relationship between depression/anxiety/stress and dysmenorrhea being an objective of the study; and published in peer-reviewed journals. The paper selection, data extraction, and quality assessment of selected studies will be performed independently by two researchers, and disagreements will be resolved through discussions. The Newcastle-Ottawa Quality Assessment Scale will be used to assess the quality of selected studies. A quantitative synthesis will be performed using the Preferred Reporting Items for Systematic Reviews and Meta-Analyses (PRISMA) via the STATA software, if retrieving enough number of studies with no severe methodological heterogeneities. Otherwise, qualitative synthesis will be used to report the findings.

**Discussion:**

To the best of our knowledge, this will be the first systematic review on this topic. Performing an inclusive search in major databases over a wide timescale is one key strength of the proposed study and will maximize the coverage of the original research studies on this topic. Results of present study are expected to lead to deeper understanding the relationship between common mental health conditions and dysmenorrhea.

**Systematic review registration:**

PROSPERO CRD42018102199

## Background

Dysmenorrhea (i.e., painful menstruation) refers to uterine cramp just before or during menstruation [1] and is the most common cause of pelvic pain and menstrual dysfunction among women in the reproductive age [2]. If menstrual cramps are developed without any pelvic pathology, they are classified as primary dysmenorrhea, which almost always occurs in women aged 20 years or younger. However, menstrual pain due to illness or pelvic pathology such as endometriosis is a secondary dysmenorrhea, which is common in women over the age of 20 years [3, 4].

The prevalence of dysmenorrhea varies greatly across different countries (16–91% [5–7]; of which, 2–40% report as being moderate to severe [4, 8]. Dysmenorrhea is one of the most common menstrual disorders, and if accompanied by symptoms such as headache, fatigue, nausea, vomiting, diarrhea, impatience, chills, and muscle cramps can disrupt the quality of life and social activities of young women [9, 10]. Primary dysmenorrhea is more common and has good prognosis. However, it still affects the quality of life because 3–33% of women experience severe pain lasting for 1–3 days during each monthly menstrual cycle [11]. In severe cases, dysmenorrhea can cause inability to function and absence from education and/or occupation [12]. Because of painful menstrual cramps, approximately 1% of women of reproductive age are unable to do their job due to severe dysmenorrhea for 1 to 3 days each month, and approximately 14% of girls are absent from education (e.g., school, college) for a day or two each month [13, 14], totaling 140 million hours of absence a year [5]. Dysmenorrhea can also impact negatively on daily activities, lower education performance at puberty, and lead to poor sleep quality, and have negative effects on mood resulting in anxiety and depression [11, 15, 16]. Dysmenorrhea has always been considered as having socioeconomic impacts because of the increased need of medical care and associated medical costs, as well as decreased women’s effectiveness in day-to-day tasks [4].

Therefore, more attention is required to address the causes and factors influencing the development and exacerbation of dysmenorrhea. Increased release or imbalanced levels of prostaglandins (especially PGF2a and PGF2) have been reported as the main cause of dysmenorrhea and are associated with increased uterine muscle tone, increased uterine contraction, decreased uterine blood flow, and increased sensitivity of peripheral nerves [14, 17]. Prostaglandins mediate inflammatory and anaphylactic reactions. In the uterus, prostaglandins are critical to vascular tone, normal blood clotting, and orchestrating labor and parturition [18].

The relationships between the severity of dysmenorrhea and several factors including age [19–22], marital history [23–25], body mass index [14, 20, 21], low socioeconomic status [19, 20, 22, 24, 26], smoking [17, 19, 23, 26], family history of dysmenorrhea [10, 27], and alcohol consumption [19, 23] have been well investigated. However, in some cases, inconsistent results have been reported. In addition to the aforementioned factors, the association between psychological factors and severity of dysmenorrhea has been described [28]. Psychological disorders such as depression, stress, and anxiety are reported as important factors associated with dysmenorrhea and menstrual disorders [17, 28, 29].

Psychological disorders such as depression, anxiety, and stress might have a bidirectional association with dysmenorrhea. In other words, experiencing monthly repeated menstrual pain might increase the risk of experiencing depression, anxiety, or stress and vice versa. In some cases, having these psychological disorders exacerbates the severity of menstrual pain [24, 30]. Experiencing depression and dysmenorrhea simultaneously might enhance the perception of pain severity and reduce the response to medication [13, 28]. Psychological distress and increased sensitivity to pain can cause idiopathic pain disorders [31]. In a critical review in 2015, Iacovides et al. proposed that patients with severe dysmenorrhea have increased pain sensitivity which cannot be explained by increased prostaglandin production alone [1]. The probable alternative explanation is central sensitivity to pain [32], which is an abnormal pain mechanism with intensified peripheral response to pain [33]. The cyclic nature of menstruation and the experience of repeated uterine inflammation raised the hypothesis that dysmenorrhea might be due to increased sensitivity to pain [34, 35]. Therefore, experiencing dysmenorrhea not only has a physical aspect but can have a psychological aspect which influences the development of psychological distress but which can also be influenced by these conditions [36]. Dysmenorrhea can be a stressor and aggravate the symptoms of depression and anxiety [37]. Evidence suggests a probable association between psychological distress and dysmenorrhea, but the underlying mechanism needs further elaboration [38].

In a previous systematic review, Latthe et al. reviewed the risk factors for chronic pelvic pain including dysmenorrhea, dyspareunia, and non-cyclical pelvic pain. They found that women with pelvic pain had more psychological disorders (depression, anxiety, neuroticism, and somatisation) than pain-free controls [23]. Although Latthe et al. found that women with dysmenorrhea had 2.77 times more chance of experiencing anxiety (95% CI 0.67–11.49) and 2.59 times higher chance of depression (95% CI 0.96–2.83), these was not statistically significant. Also, the type of dysmenorrhea (primary and secondary) was not addressed in meta-analysis. Another recent systematic review by Bajalan et al. showed the possible association between primary dysmenorrhea and depression/anxiety [39] but only investigated the evidence regarding the association between mental health attributes and primary dysmenorrhea. Therefore, the reviewing of only two mental health conditions and restricting the type of dysmenorrhea to primary cases were limitations. In previous systematic reviews [23, 39], the association of dysmenorrhea and depression, anxiety, and stress was not specifically studied, and no conclusive results were reached. The present systematic review is designed to elaborate on these associations with an updated search via a wide range of databases considering more specific outcomes. This systematic review aims to answer the following questions:

- Is there any association between depression or depressive syndromes/anxiety/stress and dysmenorrhea?
- To what extent are findings from the previous literature heterogeneous i.e., (inconsistent)?
- What are the possible sources of heterogeneity between previous studies?

## Methods

### Review preparation and registration

The protocol of this systematic review was prepared according to the PRISMA-P guidelines [40]. This study has been pre-registered in the International Prospective Register of Systematic Reviews (PROSPERO) under the reference number of CRD42018102199, which is available at: https://www.crd.york.ac.uk/prospero/display_record.php?RecordID=102199. Given that this is a systematic review and meta-analysis study of published studies, ethics approval and informed consent are unnecessary.

### Inclusion criteria

#### Type of studies

Studies will comprise those with a descriptive design including cohort, case-control, and cross-sectional that were conducted between January1990 and December 2019 and published in the English language. The relationship between depression, anxiety, or stress and dysmenorrhea will have been reported as an outcome of the study. The studies will have been published in peer-reviewed journals. Studies other than original empirical papers including systematic reviews, letters to the editor, single case reports, and case series will be excluded from the present review.

#### Types of participants

Studies will be selected with no limitation regarding participants’ age ranges, type of dysmenorrhea, menstrual irregularity, contraception method, or any chronic disease.

### Primary outcome

The association between dysmenorrhea with depression, anxiety, and stress will be considered the primary outcome of the present review.

### Potential confounding variables

Other characteristics include age, marital status, menstrual characteristics (such as menstrual regularity, duration, or amount of bleeding), family history of dysmenorrhea, parity, type of dysmenorrhea, contraception methods, existence of chronic disease, diagnostic methods for depression, anxiety or stress, study design, and study quality will be considered as potential confounders. These variables will be used for subgroup analysis.

### Search strategies for the identification of studies

Online databases such as PsycINFO, Scopus, PubMed, Science Direct, ProQuest, ISI Web of Knowledge, and Embase will be searched using keyword and MeSH terms. The target is to retrieve papers published from January 1990 to December 2019. The reference lists of all included studies will be screened for further eligible studies. Final search terms will be extracted from published reviews, primary studies, PubMed Medical Subject Headings (MeSH), and EMBASE Subject Headings (EMTREE). No limitations will be considered during the search process. The search strategy will be built upon combining the main key elements (PECO elements) [population, Exposure, comparison and outcomes] identified to answer the research question nested through Boolean operators AND/OR generating the search strings to be implemented in the scientific databases. In addition, the search syntax will be adapted for each database according their advanced search guidelines.

### Data collection and analysis

#### The selection of relevant studies

The title and abstract of all papers retrieved during the electronic or manual search process will be scrutinized using the inclusion criteria. The full texts of relevant papers will be examined based on the aforementioned criteria. These processes will be performed independently by two authors, and disagreements will be resolved through discussions. The viewpoints of an external researcher will be used if disagreements cannot be resolved through these discussions. If required, the authors of studies, whose results are published on posters, will be emailed and will be requested to send the full texts.

#### The risk of bias (quality) assessment

In the present study, the Newcastle–Ottawa Quality Assessment Scale (NOS) will be used. The NOS can be used for both case-control and longitudinal (prospective) studies. Cross-sectional studies are evaluated as case-control studies. The NOS evaluates three quality parameters: selection, comparability, and outcome. This scale is divided into eight specific items, which slightly differ when scoring case-control studies and longitudinal studies. Each item on the scale is scored with 1 point, except for the comparability, which can be adapted to the specific topic of interest to be given up to 2 points. Therefore, the maximum score for each study is 9. Any study less than 5 points is identified as high risk of bias [41]. All stages of quality assessment will be performed independently by two researchers, and the disagreements will be resolved through discussions. If needed, a third researcher will be sought if the disagreement cannot be resolved.

#### Data extraction and management

After screening and examining the quality of selected studies, data will be extracted and recorded in pre-designed forms. Data will be extracted independently by two researchers. The following data will be extracted from each study:

1. First author’s name, country in which the study was conducted, sample size, year(s) of data collection, participants’ age, type of dysmenorrhea, diagnostic measure of depression, status of depression, diagnostic measure of anxiety, diagnostic measure of stress.
2. Raw data to calculate odds ratios. If this is not possible to extract data of 2 × 2 tables for such a purpose, the parameter estimate of the unadjusted association between dysmenorrhea and depression (with 95% CI if available) will be used.

The corresponding authors of potentially eligible studies will be contacted, if duplicated studies with the same methodology and findings are retrieved and if the data for the primary outcome is missing. The authors will be contacted three times, and if no response is received, the study will be excluded. In order to manage data, the Endnote software will be used to import citations and identify duplication. Following this, extracted data according to aforementioned forms will be entered to an Excel file. Finally, the data will be exported to STATA for final synthesis.

#### Strategy for data synthesis

A quantitative synthesis using the STATA software (version 13) will be conducted. Meta-analysis will be conducted if enough studies are retrieved through the search strategy, and there is no severe methodological discrepancy among retrieved studies. To choose the best model of meta-analysis, two models of meta-analysis will be conducted for the outcomes, including the fixed-effect model and the random-effect model. A fixed-effect model will be used with the assumption that the studies are sampled from the populations with the same effect size. The Mantel-Haenszel method as an adjustment to the study weights will be used according to the in-study variance. If the studies are taken from the populations with variable effect sizes, the random effect model will be used, which calculates study weights both from in-study and between-study variances, taking into consideration the extent of variation or heterogeneity [42]. According to data abstracted from retrieved studies, the best-fitting model will be selected. Effect sizes expressed as odds ratios (for categorical data) and weighted mean differences (for continuous data) and 95% CIs will be calculated for analysis. Heterogeneity will be assessed statistically using the standard *χ*^2^ test.

The random-effect model is more appropriate when heterogeneity is present. For each model, the between-study heterogeneity will be estimated using the Q Cochrane statistic. Additionally, the level of heterogeneity will be estimated using the *I*^2^ index. *I*^2^ < 25% will be considered as mild heterogeneity, 25% < *I*^2^ < 50% will be considered as moderate heterogeneity, and 50% < *I*^2^ < 75% will be considered as severe heterogeneity [43]. In case of severe heterogeneity, subgroup analysis or meta-regression will be used. Meta-regression is an extension to subgroup analyses that allows the effect of continuous, as well as categorical, characteristics to be investigated, and in principle allows the effects of multiple factors to be investigated simultaneously (although this is rarely possible due to the inadequate numbers of studies) [44].

If the number of studies allows further stratification (and to assess the potential sources of heterogeneity), sub-analysis will be conducted for age, type of dysmenorrhea, menstrual characteristics, family history of dysmenorrhea, parity, study size (large vs. small studies), geography (country/region of residence), risk of bias (high vs. small risk of bias), and instrument used to diagnose depression (or depressive syndrome), anxiety, and stress. Also, type of study will be used as subgroup analysis. Then, pooled effect size based on subgroup analysis will be reported. For the evaluation of quality analysis and investigating the effect of the quality of original studies on the final results, the effect of study quality scores on key measurement will be assessed utilizing meta-regression or subgroup analysis according to the number of eligible studies.

Publication bias or reporting bias will be assessed using the funnel plot as well as Begg’s and Eggers’ test. If considerable publication bias is detected, the correction of final results will be conducted using the Fill and Trim method. If meta-analysis is not possible, the qualitative synthesis will be used to report the findings.

## Discussion

Dysmenorrhea is one of the most frequent discomforting gynecological conditions influencing the quality of life and social activities among women [1, 4]. Several factors can affect primary dysmenorrhea and its incidence and severity including mental health [23, 39]. Therefore, this systematic review will investigate the association between primary dysmenorrhea and psychological distress including depression, anxiety, and stress. The present systematic review will have some major strengths. To the best of our knowledge, it will be the first systematic review with meta-analysis on the topic. Performing an inclusive search in major databases across a 30-year timescale will maximize the coverage of the original research studies on this topic. Doing comprehensive subgroup analysis is expected to lead to better investigation of the factors influencing the association of dysmenorrhea with psychological distress. Results of present study are expected to provide a deeper understanding the relationship between common mental health conditions and dysmenorrhea. Such understanding will provide a better clinical insight to treat dysmenorrhea.

## Data Availability

Not applicable.

## Abbreviations

CI: Confidence intervals
GRADE: Grading of Recommendations Assessment, Development, and Evaluation
NOS: Newcastle–Ottawa Quality Assessment Scale
PRISMA: Preferred Reporting Items for Systematic Reviews and Meta-Analyses
PRISMA-P: Preferred Reporting Items for Systematic Review and Meta-Analysis Protocols
PECO: Papulation, Exposure, Comparison, Outcome
